# Acoustic Scattering in Flexible Waveguide Involving Step Discontinuity

**DOI:** 10.1371/journal.pone.0103807

**Published:** 2014-08-01

**Authors:** Muhammad Afzal, Rab Nawaz, Muhammad Ayub, Abdul Wahab

**Affiliations:** 1 Department of Mathematics, Quaid-i-Azam University, Islamabad, Pakistan; 2 Department of Mathematics, COMSATS Institute of Information Technology, Wah Cantt, Pakistan; Washington State University, United States of America

## Abstract

In this paper, the propagation and scattering of acoustic waves in a flexible wave-guide involving step discontinuity at an interface is considered. The emerging boundary value problem is non-Sturm-Liouville and is solved by employing a hybrid mode-matching technique. The physical scattering process and attenuation of duct modes versus frequency regime and change of height is studied. Moreover, the mode-matching solution is validated through a series of numerical experiments by testifying the power conservation identity and matching interface conditions.

## Introduction

The noise control problem is a subject of technological and scientific concern in the modern industrialized society. Frequent sources of unwanted noise such as vehicle, aero-engines and heating, ventilation, and air conditioning (HVAC) systems contribute a lot in environmental nuisance. In particular the noise is generated by the mechanical devices like combustion engine or fans etc. which propagates through the networks of ducts to the outside world.

In order to minimize the transmission of such unwanted noise the sound absorbent materials, acoustic lining or silencers and novel geometrical designs are significantly used [1]–[13]. The principle objective of this article is to analyze the reflection and transmission of fluid-structure coupled waves in a wave-guide consisting of elastic plate and membrane bounded ducts separated by a vertical rigid strip. Precisely, we aim to establish and analyze scattered field at the planar junction by means of the conditions of continuity for pressure and normal velocity when the elastic plate edges are clamped or pin-jointed to the vertical strip.

The solution to the analogous problems with continuous geometry are usually tractable by means of Wiener-Hopf techniques. However, the envisaged model problem involves discontinuity in geometry at the junction of two wave bearing ducts, thereby impeding the use of a classical Wiener-Hopf technique which is inappropriate for the problems involving the discontinuity in geometry or a material property [14], [15]. Nonetheless, hybrid mode-matching techniques with suitable orthogonality relation (OR) not only render a solution of the problem but also provide the physical insight of the underlying phenomena.

Initially mode-matching technique was introduced to solve the canonical problems associated with the Laplace or Helmholtz operators with imposed Dirichlet, Neumann or impedance type boundary conditions. Classically, the underlying eigen-systems are Sturm-Liouville (SL) and standard orthogonality relations lead to the solution of the problem. However, for the problems involving second or higher order boundary conditions the eigen-system becomes Non-SL and appearing modes do not satisfy the orthogonality conditions. Lawrie and Abrahams [16] developed a new form of orthogonality relations for Non-SL systems and later on Lawrie [17] stated some analytic properties of the orthogonality relations for convergence of the system. The comprehensive historic prospects of such relations have been comprehensively accounted for example in [18] and reference cited therein. The corresponding eigen-values are the roots of the dispersion relations and can be found numerically. Since then similar type of orthogonality relations have been exploited in literature to deal with assorted physical situations, see for instance [19]–[22].

In this investigation, two incident duct modes, namely structure-borne and fluid-borne modes, are considered in an elastic plate bounded duct. The boundary value problem is reformulated in a non-dimensional form with respect to length 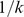 and time 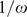, where 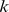 represents the wave-number. At matching interface, the incident modes scatter into the model spectrum of reflected and transmitted modes. The standard procedure of separation of variables is then used to express the reflected and transmitted potentials in an eigen-expansion form. Since the boundary conditions involve higher order derivatives, the eigen-system of the duct region is non-Sturm-Liouville. The generalized orthogonality relation such as those used in [14], [15] enables the continuity conditions of pressure and the normal velocity to recast the problem in the infinite system of linear algebraic equations, which are truncated and solved simultaneously. The truncation of higher order modes and the use of appropriate edge conditions finally lead to the solution of the scattering problem. The clamped and pin-jointed edge conditions are invoked at the junctions of elastic plate and membrane with the vertical strips.

In numerical section, we have debated the power distribution for elastic plate and membrane bounded ducts for both the fundamental and secondary mode incidents. Whereas Warren et al. [14] discussed the same for rigid and membrane bounded ducts only for the case of fundamental mode. They opted to validate the mode-matching solution with the Wiener-Hopf solution for planar structures. But in this analysis we have validated the results by plotting the continuity conditions at matching interface as well as achieved the conserve power identity. Such an approach of validating the results can be seen in [3] while the results for both the fundamental and secondary mode incidents were also formulated and employed in [15]. It is worthwhile declaring that the results for different wall conditions cannot appear as a general or special form of each other. However a consistency in the behavior of power distribution is observed throughout.

The rest of the investigation is arranged in the following order. The next section is dedicated to formulate the boundary value problem governing the wave propagation in the wave-guide. A mode-matching solution is constructed in the subsequent section. The edge conditions and their implications on the scattering pattern are then discussed. Graphical results are presented to discuss the distribution of power against frequency and vertical discontinuity. Finally, the important contributions of the investigation are summarized.

## Mathematical Formulation

Consider a two dimensional infinite wave-guide consisting of two semi infinite duct sections with different heights. The lower wall of both duct sections is assumed to be acoustically rigid. The upper surface of inlet duct section consists of an elastic plate whereas that of the outlet duct section is a membrane. The upper surfaces of the inlet and outlet duct sections are connected by means of a vertical rigid strip and respectively meet the strip at heights 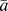 and 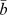 where 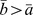. In a two-dimensional Cartesian frame of reference 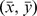 the duct sections occupy the regions 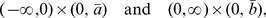
respectively. The waveguide is filled with compressible fluid of density 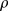 and sound speed 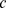.

**Figure 1:**
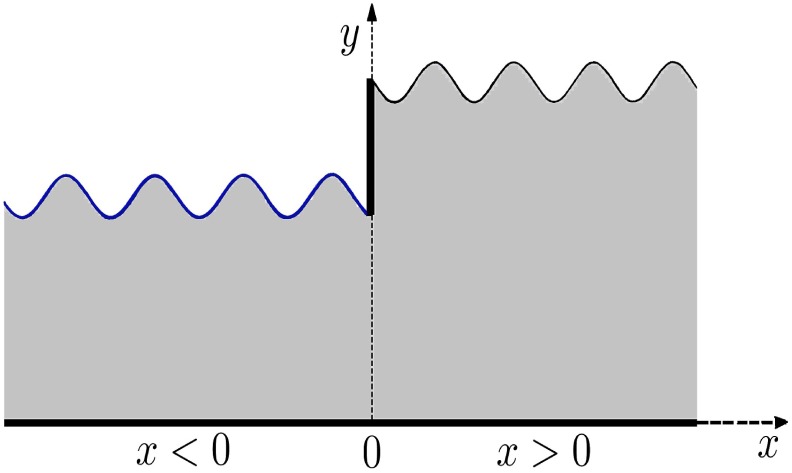
Non-dimensional geometry of wave-guide.

Throughout this work, a harmonic time dependence 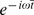 is assumed and suppressed where 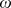 is the angular frequency in radians. The problem is non-dimensionalized relative to length and time scales 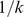 and 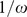 respectively by virtue of the transformation 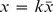 and 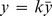 etc. The non-dimensional geometry of the problem is depicted in Figure 1.

Let 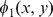 and 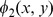 be the potential fields in the inlet and outlet duct sections respectively. The non-dimensional velocity potential 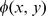 in the wave-guide can be defined as 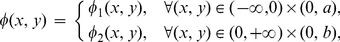
(1)which satisfies the Helmholtz equation 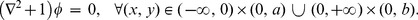
(2)


The natural conditions in non-dimensional form at lower acoustically rigid wall for both duct regions are 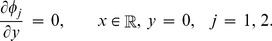
(3)


Since the upper surface of the inlet duct section comprises of an elastic plate, the boundary condition at surface 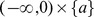 in non-dimensional form is given by 
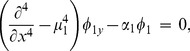
(4)where subscript 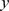 indicates a derivative with respect to 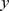, 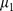 is the non-dimensional *in vacuo* plate wave-number and 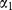 is a fluid loading parameter defined by 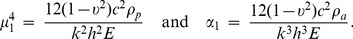
(5)


Here 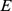 is the Young's modulus, 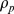 is the density of the plate, 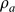 is the density of the compressible fluid and 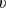 is the Poisson's ratio.

On the other hand, since the upper surface of outlet duct section is assumed to be a membrane, following non-dimensional membrane boundary condition is imposed 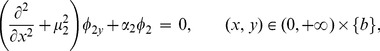
(6)where 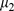 and 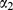 are respectively non-dimensional membrane wave-number and fluid loading parameter defined by 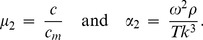
(7)


In Equations (7) above, 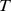 denotes the membrane tension per unit length (in the normal direction) and 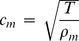 denotes the speed of waves *in vacuo* on the membrane where 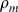 is the membrane mass per unit area.

At the matching interface, 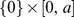 (coined as aperture), the fluid pressure and the normal component of velocity are continuous whereas the normal component of velocity vanishes on 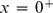, 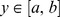. Therefore, the following continuity conditions hold: 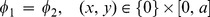
(8)and 
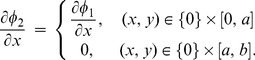
(9)


In addition, the edge conditions are applied at the points where elastic plate and membrane are joined with rigid vertical strip. These conditions not only ensure a unique solution of the boundary value problem but also describe how the elastic plate or membrane are connected to the strip. The choice of edge conditions can significantly alter the scattered field. The zero displacement (resp. zero gradient) condition at membrane edge is 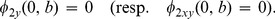
(10)


The above mentioned class of boundary value problems having wave-bearing boundaries has been discussed in detail by many researchers, see for instance [1], [3], [5], [14], [15]. The boundary conditions involve only even order derivatives in 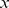 since odd order derivatives do occur in systems which are damped, and the occurrence of such derivatives significantly alters the nature of the underlying eigen-system. In particular, the dispersion relation will not be an even function of the wave number. It is not, therefore, immediately obvious that the results presented herein apply to such systems. Note also, that higher order derivatives in 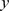 are easily removed by recourse to the governing wave equation. The underlying structure with its mathematical model is quite significant and physically admissible [2], [4], [23]. The solution to the above stated problem is presented in the next section.

## Mode-Matching Solution

Let an incident wave of an arbitrary duct mode be propagating in inlet duct section from the negative 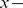
direction towards 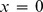. At the planar junction of ducts or discontinuity, that is, at 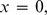 it will scatter into potentially large number of reflected and transmitted modes. The eigen-expansion form of scattered velocity potentials in duct regions take the forms 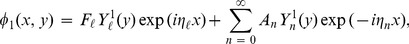
(11)and 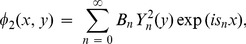
(12)where 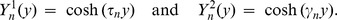



The first term in equation (11) represents the incident wave with an arbitrary forcing 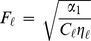 so that the incident power is unity. The counter 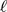 assumes values 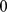 or 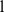 according to fundamental or secondary mode incidence respectively. The parameters 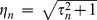 and 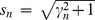 are the complex wavenumbers of 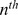 reflected and transmitted modes respectively, where 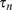 and 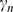 for 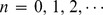 are the eigen-values of the eigen-system. The eigen-values 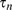 and 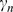 are the roots of the dispersion relations 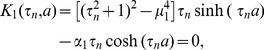
(13)and 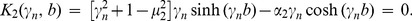
(14)


The dispersion relations (13)–(14) can be solved numerically for 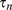 and 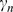 which, in turn, satisfy the following properties.

a. For each root 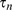 or 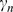, there is another root 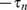 or 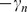.

b. There is a finite number of real roots.

c. There is an infinite number of imaginary roots.

d. The complex roots 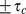 or 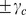 and their complex conjugates 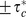 or 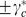 occur for some frequency ranges.

The real and imaginary roots are taken by employing a convention that the positive roots, 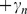, are either positive real or have positive imaginary part. They are sorted sequentially by placing real root first and then by increasing imaginary part, so that 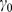 is the largest real root. For any complex root 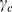 lying in the upper half of the complex 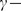
plane, the root 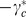 also lies in same half plane. The sequence of such pairs is taken according to the magnitude of their imaginary part, and in the order 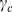 is followed by 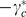. Furthermore, it is assumed that all roots have multiplicity one.

The above proposed eigen-system is non-SL system [16] and the eigen-functions 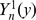 and 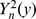 are linearly dependent [17] however satisfy the special OR. The use of ordinary orthogonality relations (ORs) is inappropriate in this case. Following the procedure devised in [14] the appropriate ORs for given eigen-system are found to be 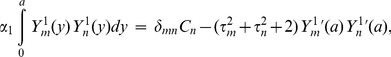
(15)and 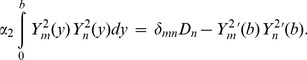
(16)


Note that 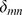 is the Kronecker's delta function and the prime indicates a differentiation with respect to 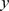 whereas 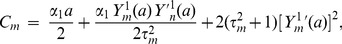
(17)and 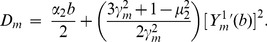
(18)


The complex amplitudes of 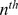 reflected and transmitted modes, 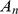 and 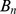, are the unknowns to be determined. The substitution of model expansion of scattered fields (11–12) into the continuity conditions (8–9) lead to an infinite system of algebraic equations thereby providing the values of 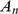 and 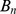. The resultant algebraic system can be solved by neglecting higher order modes corresponding to 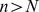 for some 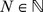. Using (11–12) into (8), the continuity condition of pressure yields 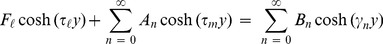
(19)


Finally, multiplying (19) with 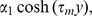 integrating over 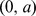 and subsequently exploiting OR (15) it is found that 
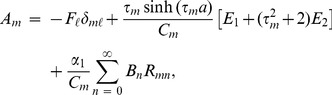
(20)where 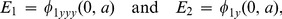
(21)and 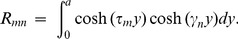
(22)


Similarly by invoking (11–12) into (9), multiplying with 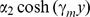, integrating from 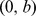 and exploiting OR (16) the expression for 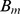 is found to be 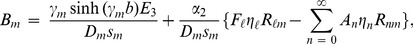
(23)where 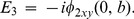
(24)


In Equations (21) and (24), the constants 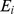 (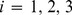) are to be precised to ensure the uniqueness of the scattering pattern and the mode-matching solution. This requires appropriate conditions at the points connecting elastic plate and membrane with vertical strip. The subsequent section is dedicated to invoke different edge conditions thereby fixing the values of these constants.

## Edge Conditions

A common assumption, when modeling wave-guide structures, is that the duct walls are clamped at the joint. In practice, however, the duct sections may be simply supported together. Therefore this section investigates different effects that arise when the edges are (a) clamped and (b) pin-jointed at the junction. The former edge conditions are characterized by zero membrane displacement and zero gradient while the latter by zero plate displacement and zero plate bending moment. A comprehensive list of appropriate edge conditions can be found, for example, in references [5], [24], [25]. As proved by Lawrie [17], for structures involving elastic plates or membranes, the number of edge conditions are half of the order of plate/membrane conditions. In fact, this imposes additional constraints on the solution to the underlying boundary value problem which also ensures the uniqueness of the solution. In the sequel, two different admissible conditions, precisely clamped edge and pin-jointed edge conditions, are considered in order to cater various industrial applications.

### Clamped edge condition

In this case the elastic plate is connected along vertical rigid strip edge in the clamped connection. The appropriate edge conditions correspond to be the zero displacement and zero gradient. That is 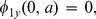
(25)and 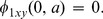
(26)


On multiplying (20) with 
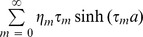
 and using edge condition (26), it is found that 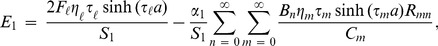
(27)where 
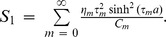
 From (21) and (25), it is obvious to find 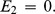 On using the zero displacement edge condition, (10) results 
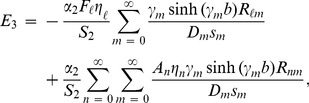
(28)where 
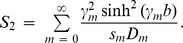


### Pin-Jointed edge condition

For the case in which the plate is pin-jointed (simply supported) along the edge 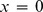, 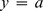. The appropriate edge conditions are 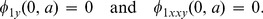
(29)


On imposing (29) in a similar fashion as for clamped edge condition, it is found that 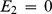 and 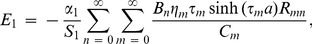
(30)where 
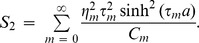
 Moreover the zero gradient 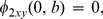
(31)is considered at membrane edge yielding 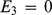.

## Numerical Results and Discussion

For the given non-SL system, (20) and (23) constitute a system of infinite number of linear algebraic equations which, together with the different values of 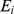 (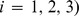 for either clamped edge or pin-jointed edge situations, is truncated and solved numerically. The numerical solution converges point-wise to the desired solution. The truncation of (20) and (23) at 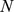 corresponds to 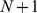 equations, where 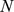 is the number of truncated modes.

In order to discuss wave propagation in similar structures as considered herein often requires the study of the power balance. There are two admissible means of energy propagation: through the fluid and along the flexible boundary. The convenient expressions for the (non-dimensional) energy flux across an arbitrary vertical strip in a duct bounded above by an elastic plate and membrane, and below by a rigid wall are given by 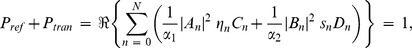
(32)where 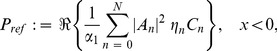
(33)gives the reflected power in inlet duct and 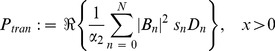
(34)shows the transmitted power to outlet duct. The power expressions (32) to (34) can be found in [18] which were also utilized by Warren et al. [14] for a membrane bounded duct. These expressions incorporate both the fluid and the structure-borne components of energy flux and can also be derived using the approach taken by Carighton and Oswell [26] together with the appropriate OR.

The dynamic interaction between a fluid and a structure is a major apprehension in many engineering problems. These problems include systems as diverse as offshore and submerged structures, storage tanks, bio-mechanical systems, ink-jet printers, aircrafts, and suspension bridges. The interaction can extremely change the dynamic properties of the structure. Therefore, it is desired to accurately model these diverse systems with the inclusion of the fluid-structure interaction. In order to see the fluid structure interaction, the fluid and structural equations need to be represented as energy equations for reflected and transmitted modes. This analysis presents a treatment of the interaction of an acoustic fluid with a flexible structures. The numerical results presented in this section consist of comparison between reflected and transmitted components of power against frequency and change of height, for both the structural-borne fundamental and the fluid-borne second mode incidence, and to validate the mode-matching technique, conditions are verified for the real and imaginary parts of pressure and velocity at the interface 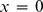.

In the sequel we assume that the inlet duct contains elastic plate of aluminum with thickness 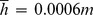 and density 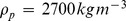. The values of Poisson's ratio and Young's modulus are taken to be 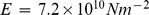 and 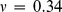 respectively; while 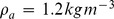 and 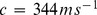. The outlet duct comprises membrane of mass density 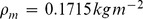 and tension 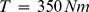.

**Figure 2:**
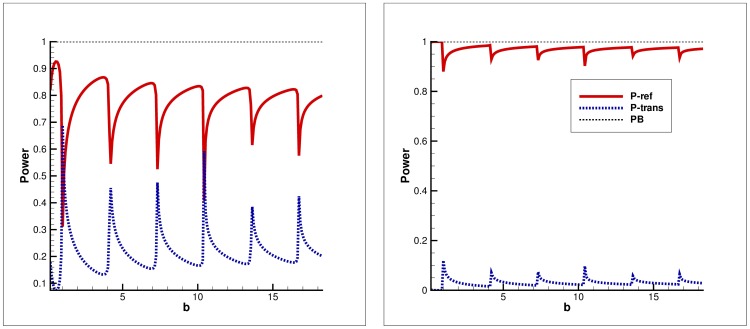
Power balance versus non-dimensional height for fundamental mode incidence. Left: Clamped edge conditions, Right: Pin-jointed edge conditions.

**Figure 3:**
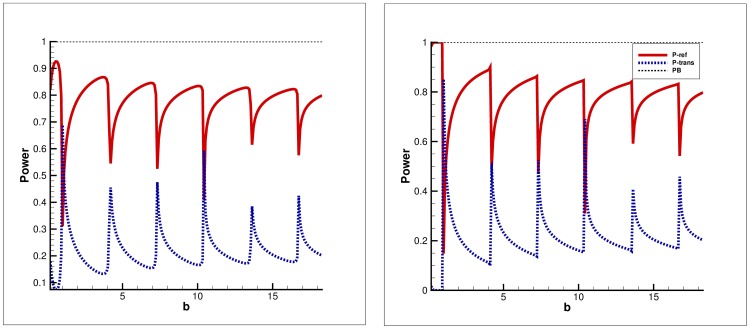
Power balance versus non-dimensional height for secondary mode incidence. Left: Clamped edge conditions, Right: Pin-jointed edge conditions.

**Figure 4:**
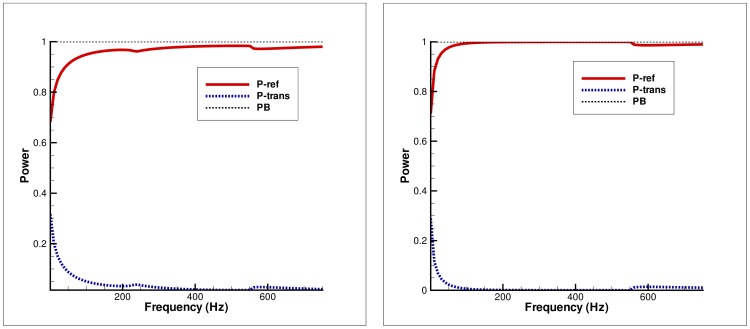
Power balance versus frequency for fundamental mode incidence. Left: Clamped edge conditions, Right: Pin-jointed edge conditions.

**Figure 5:**
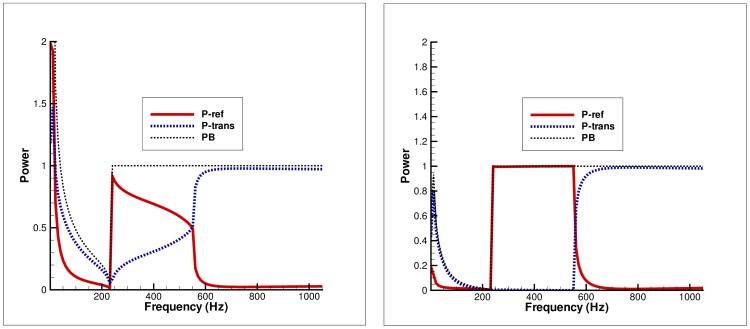
Power balance versus frequency for secondary mode incidence. Left: Clamped edge conditions, Right: Pin-jointed edge conditions.

**Figure 6:**
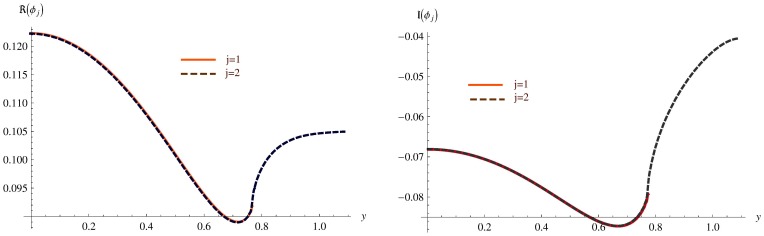
The continuity of pressure at the matching interface for 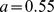 and 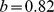. Left: Real part, Right: Imaginary part.

**Figure 7:**
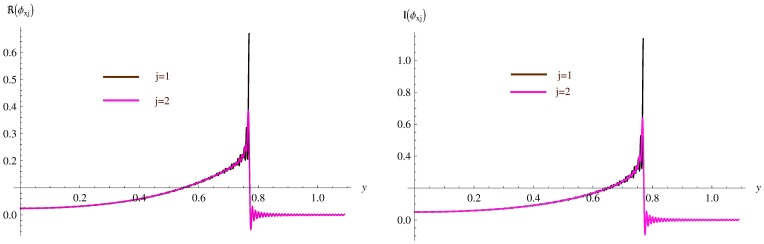
The continuity of normal velocity at hte matching interface for 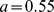 and 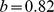. Left: Real part, Right: Imaginary part.

Figures 2–7 are delineated for two different field incidences, that is, the fundamental mode incidence and secondary mode incidence. The results show that for the fundamental mode incidence (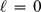) maximum of energy (in excess of 99% of energy) is carried in the plate whereas for secondary mode incident (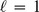) in excess of 99% of energy is in the fluid.

### Power distribution versus height of outlet duct

In Figures 2–3, the power components are plotted versus 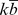 (the non-dimensional height) by fixing physical height of inlet duct at 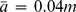 and varying the height of outlet duct from 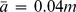 to 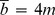.

#### Fundamental mode incidence

It is observed that for the case of fundamental mode incidence (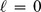), when 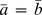 the maximum power goes on reflection for both clamped and pin-jointed conditions, where 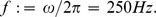 The overall trend on increasing outlet duct height is reflection over periodic fluctuation at the point where every new mode becomes propagating. It is worth mentioning that we have used rigid and the flexible walls of different conditions in the configuration of inlet and outlet ducts. Therefore the inlet duct modes and outlet duct modes are coupled due to flexible walls. For the fundamental mode incidence the maximum of the incident power goes on reflection which is consistent with available results; see for example Warren et al. [14] for rigid inlet and flexible outlet duct walls. Note that 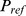, 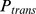 and 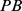 represent the reflected power, transmitted power and their sum (Power Balance) respectively.

#### Secondary mode incidence

Unlike fundamental mode incidence, when the secondary mode is incident (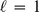), at 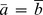 the 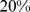 of incident power is transmitted whereas the other goes on reflection. On varying the physical height 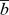 of the outlet duct section, the transmission reaches upto 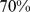 of incident power at the point where a new mode is cut-on. But once a new mode becomes propagating, reflection increases upto 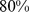 and transmission decreases inversely. The overall trend is that the reflection and transmission behave inversely for both clamped and pin-jointed edges.

### Power distribution versus frequency

Figures 4–5 depict the distribution of power in elastic plate and membrane bounded ducts against frequency values. The physical height of inlet duct is fixed at 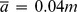 while the outlet duct achieves height 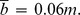


#### Fundamental mode incidence

It can be seen that for the case of fundamental mode incidence (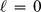), the maximum power goes on reflection for both clamped and pin-jointed edge conditions. However relatively more reflection for later edge condition along with zero gradient condition at membrane edge is observed. But the power balance identity (32) is achieved successfully in whole frequency regime for both edge conditions.

#### Secondary mode incidence

Figure 5 elucidates the power balance versus frequency in the case of secondary mode incidence (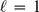).

The graph on the left in Figure 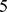 is obtained by choosing the clamped edge condition at elastic plate edge (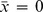, 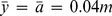) and zero displacement condition at membrane edge (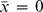, 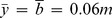), and finally plotting power components verses frequency. It can be seen that in the frequency range 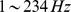 the power balance identity (32) is not achieved (dotted line) due to the cut-off inlet duct mode. At frequency 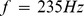, the inlet duct mode becomes propagating and the 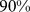 of the incident power goes on reflection which decreases steadily by increasing frequency. However at 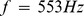 the reflected and transmitted power is distributed equally in duct regions. It is the point where the membrane bounded duct mode (outlet duct mode) becomes propagating. Once outlet duct mode is cut-on the maximum power goes on transmission whereas reflection is very small.

On the other hand, the graph on the right in Figure 5 is obtained by assuming the pin-jointed condition at elastic plate edge and zero gradient condition at membrane edge. The graph shows that as inlet duct mode is cut-on at 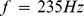, the entire incident power is reflected and consequently there is no transmission. However, once outlet duct mode is cut-on 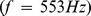 it suddenly decreases and maximum of incident power goes on transmission.

### Validation of the technique

Figures 6–7 show the continuity of pressure (8) and normal velocity (9) at the matching interface for 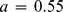 and 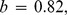 the non-dimensional heights of inlet and outlet ducts respectively.

It is clearly substantiated in Figure 6 that at matching interface, that is, 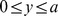, the real parts of non-dimensional pressures 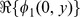 and 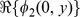 show a good agreement (see left graph in Figure 6), where 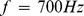. The imaginary parts behave similarly (see right graph in Figure 6).

In Figure 7, the real and imaginary parts of normal velocities 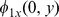 and 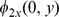 are plotted which also elucidate a very close agreement when 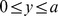.

In contrast to above discussion, the power distribution for only fundamental mode/plane wave incidence for membrane/rigid bounded duct can be seen in [14]. However the current study focuses on the elastic plate bounded inlet duct with two different incidence modes that are, fundamental mode incidence and secondary mode incidence. Though the wall conditions and the physical edge conditions are generally different yet the power distribution behavior for the fundamental mode incidence is consistent with Warren et al. [14]. Whereas for secondary mode incidence the power distribution behavior is consistent with the results presented in [15]. Therefore along with the validation of matching conditions the underlying eigen-system is consistent with that of previous studies [3], [14], [15].

### Conspectus

By virtue of the aforementioned numerical results and discussion we have the following pronouncements.

a. The numerical agreement of continuity conditions (8–9) at matching interface and validation of power balance identity (32) substantiate the validity of the mode-matching solution.

b. It is important to note that for the fundamental mode incidence the pin-jointed edges minimize the power transmission as compared to the clamped edges. However for secondary mode incidence, it increases the rates of power distribution in duct sections.

c. It is worth commenting that the choice of edge condition does not affect the attenuation of flexible duct modes. In fact, the choice of edge conditions imposed on the flexible boundaries at the junction significantly affects the transmission of energy along the duct. However, it does not affect the attenuation of flexible duct modes as can be visualized in Figures 2–7, wherein the attenuation is consistent for any selection of edge conditions.

## Conclusions

An analytic solution to scattering problem of a plane acoustic wave propagating in a rectangular waveguide involving a step discontinuity is presented. It is a well-studied phenomenon [1], [3], [4], [26] that a membrane or elastic plate attached with the mouth of an expansion chamber can effectively reduce the transmission of low-frequency noise in ducting system. The investigation was carried out to get a structure with a view to its use as a component of a modified silencer for heating ventilation and air-conditioning (HVAC) ducting systems. The model problem and the traveling wave form for the duct regions was formulated by utilizing the orthogonality relations appropriate to the eigen value problem derived through separation of variable procedure. The boundary value problem has been reduced to an infinite system of algebraic equations which requires the use of mode matching technique, which in fact, is not limited either to waveguides with planar boundaries or to two-part problems. The infinite system of equations have been solved by truncating the higher order modes and the system converged adequately. The discussion based on numerical results and physical aspects of elastic plate and membrane bounded ducts was presented in detail whereas the dimensions of the parameter were consistent with that of a typical HVAC duct. It is observed that in case of fundamental mode incidence the use of pin-jointed edge conditions contributed in minimizing the power transmission as compared to the clamped edge conditions. However, for secondary mode incidence the rates of power distribution in duct sections is increased. It is worth mentioning that the conservation of power and matching interface conditions guarantee the validity of the mode-matching solution.
